# Socioeconomic Inequalities in Human Immunodeficiency Virus (HIV) Sero-Prevalence among Women in Namibia: Further Analysis of Population-Based Data

**DOI:** 10.3390/ijerph18179397

**Published:** 2021-09-06

**Authors:** Michael Ekholuenetale, Herbert Onuoha, Charity Ehimwenma Ekholuenetale, Amadou Barrow, Chimezie Igwegbe Nzoputam

**Affiliations:** 1 Department of Epidemiology and Medical Statistics, Faculty of Public Health, College of Medicine, University of Ibadan, Ibadan 200284, Nigeria; 2 Department of Tropical Hygiene and Public Health, Medical Faculty, Heidelberg University, 69117 Heidelberg, Germany; honuoha@gmail.com; 3 Department of Economics, Faculty of Social Sciences, National Open University of Nigeria, Abuja 900211, Nigeria; charityehis2016@gmail.com; 4 Department of Public & Environmental Health, School of Medicine & Allied Health Sciences, University of The Gambia, Kanifing 3530, The Gambia; abarrow@utg.edu.gm; 5 Department of Community Health, Center of Excellence in Reproductive Health Innovation (CERHI), College of Medical Sciences, University of Benin, Benin City 300001, Nigeria; chimezie.nzoputam@gmail.com

**Keywords:** low socioeconomic, HIV/AIDS, Namibia, concentration index, inequalities, women’s health

## Abstract

Socioeconomic inequality is a major factor to consider in the prevention of human immunodeficiency virus (HIV) transmission. The aim of this study was to investigate socioeconomic inequalities in HIV prevalence among Namibian women. Data from a population-based household survey with multistage-stratified sample of 6501 women were used to examine the link between socioeconomic inequalities and HIV prevalence. The weighted HIV prevalence was 13.2% (95% CI: 12.1–14.3%). The HIV prevalence among the poorest, poorer, middle, richer, and richest households was 21.4%, 19.7%, 16.3%, 11.0%, and 3.7%, respectively. Similarly, 21.2%, 21.7%, 11.8%, and 2.1% HIV prevalence was estimated among women with no formal education and primary, secondary, and higher education, respectively. Women from poor households (Conc. Index = −0.258; SE = 0.017) and those with no formal education (Conc. Index = −0.199; SE = 0.015) had high concentration of HIV infection, respectively. In light of these findings, HIV prevention strategies must be tailored to the specific drivers of transmission in low socioeconomic groups, with special attention paid to the vulnerabilities faced by women and the dynamic and contextual nature of the relationship between socioeconomic status and HIV infection.

## 1. Introduction

Globally, human immunodeficiency virus (HIV) continues to pose a serious public health challenge. Since the epidemic started, about 76 million people have been infected, and 33 million people have died from Acquired Immunodeficiency Syndrome (AIDS)-related illnesses. The 2019 estimates show 38 million people living with HIV (PLHIV) worldwide, 1.7 million new HIV infections, and 690,000 AIDs-related deaths [1,2]. With a global prevalence of 0.7% among adults, the HIV burden varies significantly between regions and countries [1,2]. Despite global progress in the HIV response, Africa continues to bear the brunt of the epidemic, with a prevalence of 3.7% [1,2]. Sub-Saharan Africa (SSA) remains disproportionately burdened with more than two-thirds (67.4%) of the 38 million PLHIV [1,2]. In addition, SSA accounted for 57% of 1.7 million new HIV infections worldwide, and 64% of all AIDS-related deaths [1,2].

The 2016 Political Declaration on HIV and AIDS, adopted at the United Nations (UN) General Assembly High-Level Meeting on AIDS in June 2016, reaffirmed the 2030 Agenda for Sustainable Development. Seven of the seventeen Sustainable Development Goals (SDGs) including ending AIDS epidemic by 2030 (SDG 3.3), and those focused on poverty, hunger, education, gender equality, decent work, and reducing inequalities, are relevant to effective HIV response globally and within countries [3,4]. The concept of inequality among the populace is notable. The persistent high HIV prevalence and burden in SSA appears to be linked to income inequality [2]. Using data from 46 countries in SSA, the UN University World Institute for Development Economics Research (UNU-WIDER) demonstrated a positive relationship between HIV prevalence and income inequality after adjusting for differences across countries in levels of education, gender inequality, gross domestic production, and corruption [2,5]. This validates findings from other recent studies, reporting that social and economic disparities drive health inequalities across and within countries and that higher socioeconomic status is positively associated with better health outcomes [2,6,7,8,9,10,11].

The Southern Africa region appears to be the epicenter of the HIV epidemic, with estimated 54.5% of the 38 million PLHIV, 43% of all new HIV infections, and 43% of all AIDS-related deaths [1,2]. The leading countries with HIV prevalence include Eswatini (Swaziland), Lesotho, Botswana, South Africa, Zimbabwe, Mozambique, Namibia, Zambia, and Malawi, with prevalence ranging from 8.9% to 27% [1,2]. HIV prevalence in Namibia is among the highest in the world. Currently, Namibia has HIV prevalence of 11.5%, with approximately 210,000 PLHIV and 3000 AIDS-related deaths in 2019 [2].

Women are disproportionately affected by HIV. In a recent report, women and girls accounted for 63 percent of all new HIV infections in SSA in 2020 [1]. In Namibia, HIV prevalence is higher for women (16.9%) than men (10.9%) [12]. Unequal gender norms limit women’s and girls’ voices, limit their access to education and economic resources, and inhibit civic participation, all of which contribute to women’s increased HIV risk in high-HIV-prevalence environments [2]. Women are more vulnerable or most at-risk of HIV transmission and therefore become a key population of interest for HIV interventions. It is against this backdrop that this study used women as study participants. 

Many HIV-related epidemiological research studies have concentrated on absolute poverty as a risk factor for HIV infection; however, studies from SSA have recently indicated that socioeconomic disparity is a bigger driver of HIV transmission than absolute poverty or wealth. Despite Namibia’s high overall HIV prevalence, wide disparities in sub-national HIV prevalence, and the highly socioeconomically heterogeneous nature of its states, none of the few studies on socioeconomic inequality as a driver of HIV transmission in SSA have extensively used Namibia as a case study. Therefore, the goal of this study was to look into the differences in HIV prevalence among Namibian women based on socioeconomic status.

## 2. Materials and Methods

### 2.1. Ethical Consideration

We used population-based secondary datasets from the public domain/online that were stripped of any identifier information for this investigation. MEASURE Demographic and Health Survey (DHS)/Inner City Fund (ICF) International gave the authors permission to use the data. The DHS Program adheres to industry norms for preserving the privacy of respondents. ICF International assures that the survey complies with the Human Subjects Protection Act of the United States Department of Health and Human Services. This study did not require any additional approvals. More details about data and ethical standards are available at http://goo.gl/ny8T6X (accessed on 15 June 2021). 

### 2.2. Data Source

We used nationally representative cross-sectional data from the 2013 Survey of Namibia DHS. Data on 6501 women aged 15–64 years were extracted from the study individual-level DHS survey and analyzed. These data linked survey responses with HIV test results from biomarker data. 

The Namibia DHS began in April 2012, and data gathering took place from May to September 2013. The survey’s general goal was to collect demographic, socioeconomic, and health data that might be used for national and regional policymaking, planning, monitoring, and evaluation.

The specific survey’s goal was to extract reliable data on HIV/AIDS, to explore the current prevalence, and to examine if progress on the issue over time could be determined. Details of the DHS sampling procedure have been previously reported [13].

### 2.3. Variables Selection and Measurement

#### 2.3.1. Outcome 

The dependent variable was a dichotomous indicator of HIV status positivity: a value of 1 or 0 indicated whether a respondent was seropositive (1) or negative (0), respectively.

The status of the women was determined by taking a blood sample from each of them. This study looked into the HIV status of women (positive vs. negative).

#### 2.3.2. Explanatory Factors

The inclusion of these independent variables was based on the outcome of factors associated with HIV from previous studies [14,15,16,17,18]. The explanatory factors are presented in Table 1.

#### 2.3.3. Socioeconomic Variables

In this study, we extracted women’s education and household wealth index variables as socioeconomic factors. DHS computed a household wealth index using principal component analysis (PCA). These were the variables extracted for socioeconomic measurements. Women’s education and household wealth quintiles were utilized as socioeconomic status indicators in this study. Inequalities in HIV prevalence were investigated as a result of household wealth and women’s educational level. Previous studies [19,20,21,22,23] also used women’s education, household wealth, or both while investigating for socioeconomic inequalities, as these two indicators were identified as the most important measures of socioeconomic status. Women’s education was categorized into groups (no formal education, primary, secondary, higher).

The household wealth index was calculated as a cumulative composite of each polled household’s living standard. It was calculated using data that were simple to obtain on each of the surveyed household’s specified assets. Bicycles and televisions were among the selected assets, as were the materials used to build or construct the houses, the sort of water available to the families, and their sanitary facilities. The wealth index for each household was calculated using principal component analysis and placed on a continuous relative wealth scale. DHS divided the questioned families into five wealth quintiles in principle, using PCA to compute the household variables. PCA has been established and validated as a tool for explaining how a population’s socioeconomic status is differentiated within that population. It has also been used to cut down on the number of variables in a batch of data [24]. Each household’s *z*-scores and factor loadings (factor coefficient) was determined. The loadings were multiplied by the indicator values of each household and then summed to provide the wealth index value for each household. With the standardized z-scores, the overall assigned scores of the poorest/poorer/middle/richer/richest groups were disentangled [12]. 

### 2.4. Analytical Approach

To account for sampling design, the Stata survey module (‘svy’) command was utilized. Percentage was used in the univariate test. Socio-economic inequalities were studied using the Lorenz curve and concentration index for HIV prevalence. The concentration index value is positive when HIV prevalence is higher among high socioeconomic classes. When the concentration index value is negative, it suggests that HIV prevalence is high among poor socioeconomic groups. For stratified analyses, explanatory variables were used. In HIV prevalence, the concentration index was utilized to compute the contrast [23,25]. Statistical significance was determined at *p* < 0.05. Stata version 14 (StataCorp., College Station, TX, USA) was used for data analysis.

## 3. Results

The weighted HIV prevalence was 13.2% (95% CI: 12.1–14.3%). In Table 2, the results showed increasing HIV prevalence as women advanced in age. The prevalence was higher among those in rural residence, among female headed households, among women with no media use (newspaper/magazine, radio and TV), those not covered by health insurance, and those formerly in a union and not employed, respectively. See Table 2 for the details.

In Table 3, the HIV prevalence among the poorest, poorer, middle, richer, and richest households was 21.4%, 19.7%, 16.3%, 11.0%, and 3.7%, respectively. The results show a decreasing pattern of HIV prevalence across women’s characteristics. HIV prevalence were higher among women from low household wealth quintiles. See Table 3 for the details.

In Table 4, approximately 21.2%, 21.7%, 11.8%, and 2.1% HIV prevalence was estimated among women with no formal education and primary, secondary, and higher education, respectively. The educated women had the lowest HIV prevalence, and this pattern was consistent across women’s characteristics. See Table 4 for the details.

**Figure 1:**
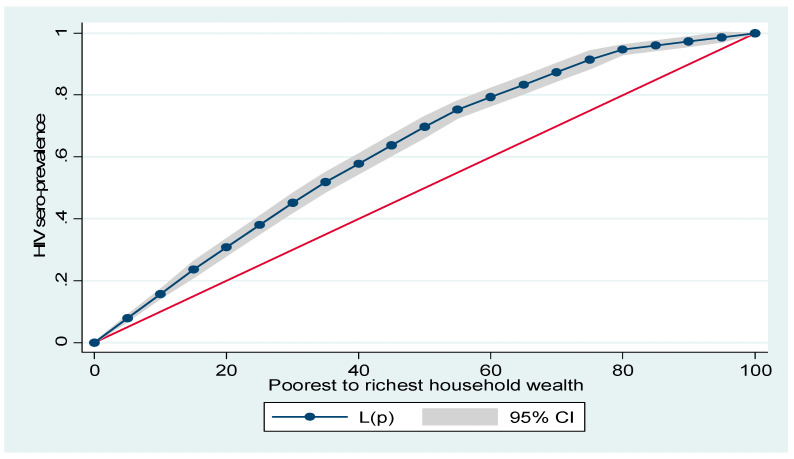
Lorenz curve for HIV prevalence by women’s household wealth.

**Figure 2:**
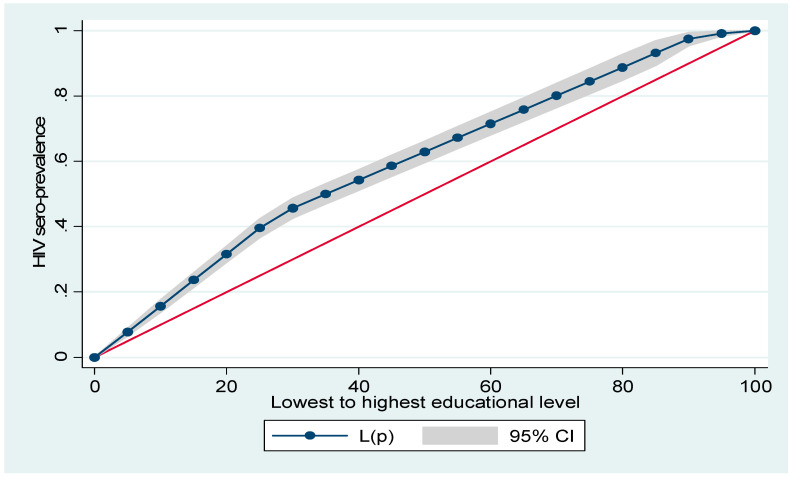
Lorenz curve for HIV prevalence by women’s education.

Figure 1 and Figure 2 show household wealth and women’s education inequalities for HIV prevalence in Namibia. A higher degree of inequalities is confirmed by how far the curves sag away from the line of equality (that is the red diagonal line). Figure 1 and Figure 2 show that women from poor households and those with low formal education had a higher HIV prevalence, as the line of equality sags above the diagonal line respectively.

Table 5 shows the results of household wealth and women’s education inequalities for HIV prevalence in Namibia. Overall, women from poor households (Conc. Index = −0.258; SE = 0.017) and those with low formal education (Conc. Index = −0.199; SE = 0.015) had a higher concentration of HIV infection. Across the levels of women’s characteristics, there was higher HIV prevalence among the poor. Similarly, women with low formal education status had significantly higher HIV prevalence across the levels of explanatory factors. See Table 5 below for the details.

## 4. Discussion

We found a high HIV prevalence among Namibian women (13.2 percent). This is in line with reports of HIV prevalence in the Southern Africa region, where HIV prevalence has remained the highest worldwide [26]. In a previous study, HIV prevalence in Namibians over the age of 12 years was 11.8 percent in 2006/7 and 14.6 percent in 2009 [27]. Similarly, there are staggering reports of HIV prevalence among the adult population in Eswatini (27.3%), Lesotho (23.6%), South Africa (20.4%), Botswana (20.3%), Zimbabwe (12.7%), Mozambique (10.3% and 12.6%), and Zambia (11.3%) [18,28]. Despite efforts to reduce HIV transmission over time, the rate of reduction in incidence, combined with existing contextual interventions, indicates that Namibia is still a long way from meeting the 2030 global targets for reducing HIV incidence and mortality. 

HIV increases when girls are denied the right to make decisions about their sexual and reproductive health and well-being. Specific intervention must target gender inequality. That is why we must strengthen our advocacy, sensitization, and behavior change communication to really discuss issues of sexual and reproductive health and how communities can best be engaged in promoting the rights of women and girls to make the right decisions regarding their sexual and reproductive health. It is possible that previous interventions have not looked at the vulnerability of women to HIV infection with a view to designing female-focused interventions.

In addition, the results show that HIV infection is more prevalent among poor and uneducated women. These findings support the current global views on the relationship between low socioeconomic status and poor health outcomes, such as HIV infection [29,30,31]. The implications of the results could be that the poor and those with low educational status may be facing the dual problems of high vulnerability and a lack of possibilities to make better sexual health choices (such as access to information on prevention, testing, and counseling for HIV infection) as a result of low socioeconomic status. High HIV prevalence has been significantly associated with relative economic prospects, referred to as “relative wealth” by Fox [31]. In a previous study using DHS data from 20 countries in SSA, Magadi [29] found similar results, where the urban poor had greater risks of HIV infection than their non-poor counterparts.

Several measures to decrease HIV infection have been prompted by the growing HIV prevalence. For example, the number of HIV-positive patients receiving antiretroviral therapy (ART) in Namibia increased from 10,200 in 2004 [32] to 166,000 in 2016 [33]. On the other hand, the country has set a target to reduce new HIV infections by 75% by scaling up evidence-based interventions such as viral suppression through ART for all PLHIV and pre-exposure prophylaxis (PrEP) in high-burden areas [34]. However, no intervention has included specific designs targeted at women with low socioeconomic status, who were identified in this study as a key population. This evidence of HIV disproportionately infecting poor and uneducated women is timely and paramount for future HIV intervention designs. For example, interventions that include women’s empowerment, decision making power, girl-child education, and women’s autonomy could positively influence health care service uptake, including HIV prevention in Namibia.

Key interventions such as free HIV screening or testing and counseling and treatment for HIV positive women should be designed and supported by the government and other stakeholders in the healthcare system. This will encourage more women, especially the disadvantaged, to participate in HIV prevention, control, and treatment practices. In addition, it will be necessary for the government to enroll low income women who are HIV positive in a special financial support scheme. Such scheme could be targeted at providing economic aid to the disadvantaged women and at reducing their HIV burden. Importantly, such strategy would be useful in creating a registry for HIV positive women, which would serve as an avenue for enrolling them for ART, following-up their progress, and reducing the HIV transmission rate. Furthermore, specific messages targeted to improve awareness and knowledge of HIV among low income women, the uneducated, or those in hard-to-reach communities would be helpful in the fight against HIV.

### Strength and Limitations

The findings of this study can be applied to all women in Namibia because they are based on nationally representative data. However, due to the use of secondary data, it was impossible to quantify potential covariates such as the use of alcohol, hard drugs, and engagement in extramarital relationships, as well as other endogenous factors. Under-representation of low income women is probable in the DHS dataset. It is possible that low income women were under-represented in the survey, as some face illiteracy and perhaps could not fill out the survey or may have limited ability to accurately report their circumstances. Moreover, recruitment of the homeless or other vulnerable groups could be a major factor responsible for the under-representation of low income women. Additionally, DHS did not collect data on household income or expenditure, which are the traditional indicators used to measure wealth. The assets-based wealth index used here is only a proxy indicator for household economic status, and it does not always produce results similar to those obtained from direct measurements of income and expenditure when such data are available or can be collected reliably.

## 5. Conclusions

This study revealed high HIV prevalence among Namibian women. Furthermore, poor and uneducated Namibian women demonstrated the highest prevalence of HIV infection. These findings add to the body of knowledge about the link between HIV and socioeconomic status. HIV prevention strategies must be targeted at the vulnerabilities faced by low socioeconomic status women and the dynamic and contextual nature of the relationship between low socioeconomic status and HIV infection.

## Figures and Tables

**Table 1:** Explanatory factors for HIV prevalence among women in Namibia.

Variable	Definition
Age	15–19, 20–24, 25–29, 30–34, 35–39, 40–44, 45–49, 50+
Residential status	urban versus rural
Region	Caprivi, Erongo, Hardap, Karas, Kavango, Khomas, Kunene, Ohangwena, Omaheke, Omusati, Oshana, Oshikoto, Otjozondjupa
Sex of household head	male versus female
Frequency of reading newspaper or magazine	not at all, less than once a week, at least once a week
Frequency of listening to radio	not at all, less than once a week, at least once a week
Frequency of watching TV	not at all, less than once a week, at least once a week
Smoking	no versus yes
Health insurance coverage	not covered versus covered
Marital status	never in union, currently in union/married, formerly in union
Participation in labor force	not employed versus employed

**Table 2:** Distribution of HIV prevalence across women’s characteristics in Namibia.

Variable	*n* (%)	HIV Sero-Prevalence, %
Age		
15–19	627 (9.6)	2.6
20–24	1118 (17.2)	2.9
25–29	1072 (16.5)	9.0
30–34	924 (14.2)	16.9
35–39	866 (13.3)	23.0
40–44	758 (11.7)	24.0
45–49	592 (9.1)	19.8
50+	544 (8.4)	16.5
Residential status		
Urban	3487 (53.6)	11.2
Rural	3014 (46.4)	16.5
Region		
Caprivi	406 (6.3)	24.1
Erongo	630 (9.7)	7.9
Hardap	372 (5.7)	9.1
Karas	577 (8.9)	10.9
Kavango	507 (7.8)	16.2
Khomas	693 (10.7)	7.2
Kunene	396 (6.1)	7.1
Ohangwena	475 (7.3)	19.6
Omaheke	392 (6.0)	9.4
Omusati	549 (8.4)	23.2
Oshana	550 (8.5)	17.5
Oshikoto	480 (7.4)	14.6
Otjozondjupa	474 (7.3)	12.7
Sex of household head		
Male	2840 (43.7)	10.1
Female	3661 (56.3)	16.4
Frequency of reading newspaper or magazine		
Not at all	2074 (32.0)	20.6
Less than once a week	2105 (32.4)	13.7
At least once a week	2309 (35.6)	7.4
Frequency of listening to radio		
Not at all	1072 (16.5)	16.0
Less than once a week	1515 (23.3)	15.3
At least once a week	3908 (60.2)	12.4
Frequency of watching TV		
Not at all	2950 (45.8)	19.4
Less than once a week	869 (13.4)	12.0
At least once a week	2650 (40.8)	7.8
Smoking		
No	6050 (93.1)	13.8
Yes	451 (6.9)	11.5
Health insurance coverage		
Not covered	5303 (81.6)	15.4
Covered	1197 (18.4)	6.1
Marital status		
Never in union	3183 (49.0)	10.7
Currently in union/married	2645 (40.7)	13.1
Formerly in union	673 (10.4)	30.0
Participation in labor force		
Not employed	3302 (51.1)	14.8
Employed	3164 (48.9)	12.5
Total estimates	6501	13.2

**Table 3:** Distribution of HIV prevalence by household wealth across women’s characteristics in Namibia.

Variable	Lowest (Poorest)	Second Level	Middle	Fourth Level	Highest (Richest)
Age					
15–19	3.2	3.0	2.1	2.3	2.1
20–24	6.9	3.5	3.8	1.3	1.2
25–29	18.8	10.3	11.4	5.5	3.3
30–34	20.9	31.7	22.8	12.7	2.5
35–39	29.2	33.3	27.9	20.5	5.9
40–44	41.4	33.3	32.0	20.2	5.8
45–49	37.7	34.3	16.8	18.8	3.3
50+	20.6	21.1	19.4	16.4	6.3
Residential status					
Urban	29.8	22.3	17.1	11.6	3.9
Rural	20.9	18.6	15.5	9.6	0.9
Region					
Caprivi	29.0	25.0	24.5	22.0	10.0
Erongo	33.3	13.3	16.4	11.1	2.7
Hardap	35.7	25.0	11.8	7.2	2.4
Karas	11.8	16.7	19.7	10.5	3.2
Kavango	15.6	15.7	16.2	20.3	13.3
Khomas	20.0	21.9	15.2	5.8	1.8
Kunene	4.2	3.3	13.8	7.3	5.8
Ohangwena	24.3	21.3	13.6	9.1	0.0
Omaheke	13.3	17.5	6.4	7.1	3.1
Omusati	32.2	21.6	19.5	26.1	4.4
Oshana	24.0	23.6	21.3	13.2	6.2
Oshikoto	17.0	23.4	13.1	9.5	2.0
Otjozondjupa	25.0	24.4	13.8	10.7	7.6
Sex of household head					
Male	14.2	15.7	14.3	10.4	1.6
Female	25.6	22.7	17.7	11.4	6.4
Frequency of reading newspaper or magazine					
Not at all	23.2	23.1	19.8	14.1	14.5
Less than once a week	19.7	16.0	17.1	12.2	3.8
At least once a week	13.5	17.9	10.9	8.7	2.2
Frequency of listening to radio					
Not at all	21.9	22.1	14.1	10.0	3.0
Less than once a week	23.4	19.2	18.6	11.7	4.0
At least once a week	19.9	19.1	16.0	11.0	3.7
Frequency of watching TV					
Not at all	22.1	21.0	17.7	13.4	0.0
Less than once a week	16.7	15.6	17.5	10.8	4.5
At least once a week	11.8	12.5	12.4	10.4	3.6
Smoking					
No	21.7	19.3	16.5	11.3	4.0
Yes	18.6	26.4	14.0	7.5	0.0
Health insurance coverage					
Not covered	21.3	20.0	16.5	11.5	5.3
Covered	27.3	14.8	14.1	9.0	1.9
Marital status					
Never in union	15.8	14.4	12.4	9.3	3.7
Currently in union/married	20.7	20.4	17.3	10.3	2.3
Formerly in union	38.9	42.3	34.1	23.5	10.6
Participation in labor force					
Not employed	22.7	18.1	15.9	10.0	3.0
Employed	18.1	22.8	16.9	11.6	3.9
Total estimates	21.4	19.7	16.3	11.0	3.7

**Table 4:** Distribution of HIV prevalence by educational status across women’s characteristics in Namibia.

Variable	No Education	Primary	Secondary	Higher
Age				
15–19	0.0	3.5	2.5	0.0
20–24	11.1	7.6	2.3	0.0
25–29	16.3	14.0	8.5	1.1
30–34	26.8	34.4	13.8	0.0
35–39	29.3	29.1	22.5	1.8
40–44	24.0	29.9	23.8	6.5
45–49	27.1	26.6	16.3	5.8
50+	15.3	18.8	17.4	4.2
Residential status				
Urban	27.5	19.9	10.1	1.2
Rural	17.3	22.8	14.1	5.5
Region				
Caprivi	33.3	34.6	22.0	8.7
Erongo	25.0	13.8	7.4	1.6
Hardap	18.2	18.8	6.4	3.5
Karas	10.0	17.1	10.3	0.0
Kavango	17.1	16.3	16.2	12.5
Khomas	11.1	19.1	8.0	0.6
Kunene	4.9	9.0	7.9	0.0
Ohangwena	38.2	32.0	10.8	10.5
Omaheke	18.5	6.8	8.7	0.0
Omusati	41.9	34.0	17.2	11.1
Oshana	50.0	28.2	17.8	0.0
Oshikoto	24.1	22.9	12.0	0.0
Otjozondjupa	27.5	21.0	8.9	0.0
Sex of household head				
Male	15.3	14.3	9.5	1.1
Female	27.0	27.4	13.5	3.2
Frequency of reading newspaper or magazine				
Not at all	20.4	22.2	19.6	0.0
Less than once a week	26.1	21.0	12.5	6.7
At least once a week	42.9	20.9	7.2	0.7
Frequency of listening to radio				
Not at all	19.8	22.3	13.8	0.0
Less than once a week	28.2	21.9	13.4	3.1
At least once a week	19.2	21.5	10.8	2.2
Frequency of watching TV				
Not at all	22.5	23.2	17.0	5.3
Less than once a week	17.7	20.4	10.9	2.4
At least once a week	15.9	17.1	7.6	1.7
Smoking				
No	22.3	22.3	12.0	2.2
Yes	16.4	15.9	8.4	0.0
Health insurance coverage				
Not covered	21.4	22.0	12.9	2.3
Covered	14.3	18.4	6.6	2.1
Marital status				
Never in union	26.8	20.3	8.8	1.5
Currently in union/married	16.4	17.7	12.5	2.0
Formerly in union	26.2	36.8	28.4	8.6
Participation in labor force				
Not employed	21.1	21.9	12.1	1.8
Employed	22.0	21.9	11.4	2.3
Total estimates	21.2	21.7	11.8	2.1

**Table 5:** Household wealth and educational attainment inequalities in HIV prevalence among Namibian women.

Variable	Household Wealth Quintile		Mother’s Education	
	Concentration Index (SE)	*p*	Concentration Index (SE)	*p*
Age		0.279		0.002 *
15–19	−0.085 (0.140)		−0.068 (0.106)	
20–24	−0.324 (0.098) *		−0.342 (0.077) *	
25–29	−0.284 (0.054) *		−0.168 (0.044) *	
30–34	−0.282 (0.040) *		−0.249 (0.033) *	
35–39	−0.209 (0.034) *		−0.112 (0.030) *	
40–44	−0.297 (0.035) *		−0.095 (0.033) *	
45–49	−0.338 (0.045) *		−0.185 (0.044) *	
50+	−0.165 (0.054) *		−0.056 (0.052)	
Residential status		<0.001 *		<0.001 *
Urban	−0.325 (0.026) *		−0.251 (0.022) *	
Rural	−0.149 (0.023) *		−0.120 (0.021) *	
Region		<0.001 *		0.001 *
Caprivi	−0.100 (0.049) *		−0.122 (0.040) *	
Erongo	−0.340 (0.071) *		−0.188 (0.059) *	
Hardap	−0.428 (0.088) *		−0.272 (0.077) *	
Karas	−0.288 (0.065 *)		−0.153 (0.054) *	
Kavango	0.021 (0.056)		−0.013 (0.053)	
Khomas	−0.488 (0.071) *		−0.324 (0.066) *	
Kunene	0.066 (0.103)		−0.008 (0.097)	
Ohangwena	−0.156 (0.050) *		−0.281 (0.046) *	
Omaheke	−0.272 (0.087) *		−0.170 (0.081) *	
Omusati	−0.099 (0.043) *		−0.195 (0.038) *	
Oshana	−0.194 (0.051) *		−0.198 (0.041) *	
Oshikoto	−0.193 (0.062) *		−0.216 (0.054) *	
Otjozondjupa	−0.191 (0.065)		−0.278 (0.058) *	
Sex of household head		0.109		0.276
Male	−0.286 (0.031) *		−0.176 (0.028) *	
Female	−0.228 (0.021) *		−0.211 (0.018) *	
Frequency of reading newspaper or magazine		<0.001 *		<0.001 *
Not at all	−0.083 (0.024) *		−0.028 (0.023)	
Less than once a week	−0.180 (0.031) *		−0.121 (0.023) *	
At least once a week	−0.350 (0.040) *		−0.289 (0.032) *	
Frequency of listening to radio		0.571		0.517
Not at all	−0.224 (0.039) *		−0.162 (0.036) *	
Less than once a week	−0.231 (0.034) *		−0.185 (0.030) *	
At least once a week	−0.266 (0.023) *		−0.207 (0.020) *	
Frequency of watching TV		<0.001 *		<0.001 *
Not at all	−0.084 (0.021) *		−0.086 (0.019) *	
Less than once a week	−0.211 (0.051) *		−0.187 (0.042) *	
At least once a week	−0.250 (0.035) *		−0.233 (0.030) *	
Smoking		0.019 *		0.958
No	−0.248 (0.018) *		−0.202 (0.015) *	
Yes	−0.408 (0.071) *		−0.199 (0.069) *	
Health insurance coverage		<0.001 *		<0.001 *
Not covered	−0.190 (0.018) *		−0.148 (0.016) *	
Covered	−0.446 (0.057) *		−0.304 (0.056) *	
Marital status		0.031 *		<0.001 *
Never in union	−0.207 (0.029) *		−0.245 (0.023) *	
Currently in union/married	−0.303 (0.028) *		−0.151 (0.026) *	
Formerly in union	−0.196 (0.032) *		−0.073 (0.031) *	
Participation in labor force		0.187		0.137
Not employed	−0.235 (0.023) *		−0.175 (0.021) *	
Employed	−0.281 (0.026) *		−0.220 (0.022) *	
Total estimates	−0.258 (0.017) *		−0.199 (0.015) *	

* Significant at *p* < 0.05; *SE*, standard error; *p* = comparing concentration indices across the levels of a variable.

## Data Availability

Data for this study were sourced from Namibia Demographic and Health Surveys (DHS), which are available here: http://dhsprogram.com/data/available-datasets.cfm (accessed on 15 June 2021).
